# Supplementary material for: A Polymorphism in the TMPRSS2 Gene Increases the Risk of Death in Older Patients Hospitalized with COVID-19
Source: Viruses. 2022 Nov 18;14(11):2557. doi: 10.3390/v14112557 (PMC9696669; doi:10.3390/v14112557)
Supplement: Supplementary file 1 [file viruses-14-02557-s001.zip › viruses-2020021-supplementary.pdf]

Supplementary Material

Figure S1. Allele discrimination plot of *TMPRSS2* (A) rs12329760 and (B) rs2070788 polymorphisms.

A

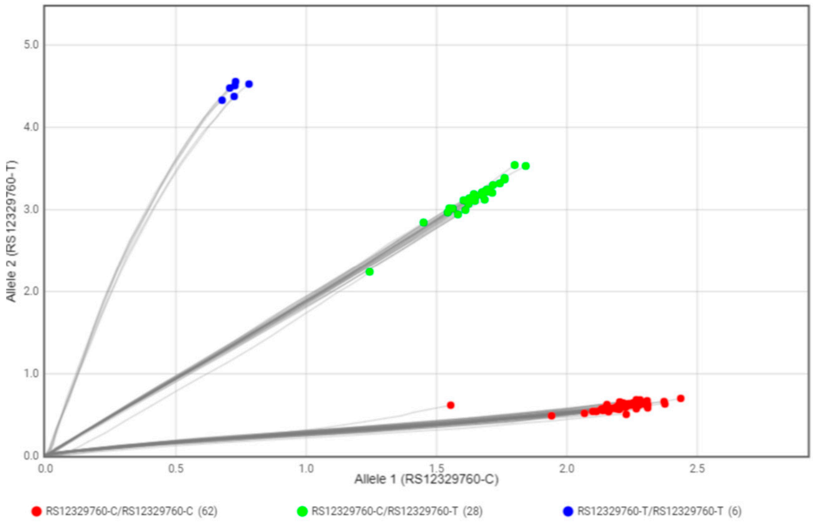

B

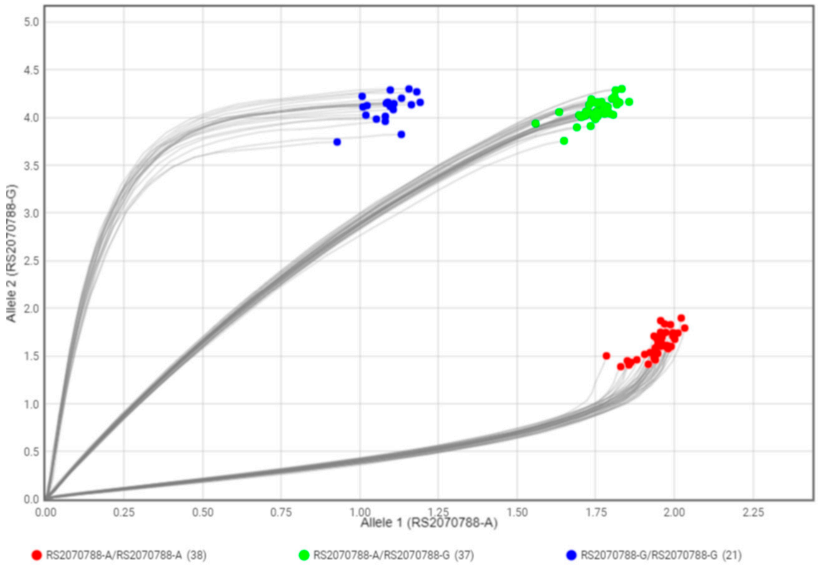

Table S1. Frequencies of *TMPRSS2* variants in Brazilian samples and in other reference populations.

SNP	Coordinate[a]	A1	SABE-Brazil[b]	MAF (1000 Genomes Project[c])					
				Global	AFR	EAS	EUR	SAS	AMR
**rs2070788**	chr21:41470061	G	0.58	0.39	0.27	0.36	0.46	0.47	0.49
**rs12329760**	chr21:41480570	T	0.20	0.26	0.29	0.36	0.24	0.23	0.15

A1: reference allele; MAF: minor allele frequency; AFR: African/African Americans; EAS: East Asians; EUR: Europeans; SAS: South Asians; AMR: Admixed Americans. [a]Human genome assembly: GRCh38, [b]SABE-Brazil - Naslavsky et al., 2022 [26], [c]1000 Genomes Project (phase 3) - 1000 Genomes Project Consortium et al., 2015 [39].
